# Digital Twins in Agriculture: Orchestration and Applications

**DOI:** 10.1021/acs.jafc.4c01934

**Published:** 2024-05-06

**Authors:** Marc Escribà-Gelonch, Shu Liang, Pieter van Schalkwyk, Ian Fisk, Nguyen Van Duc Long, Volker Hessel

**Affiliations:** 1 Higher Polytechnic Engineering School, University of Lleida, Lleida 25001, Spain; 2 ARC Centre of Excellence Plants for Space, University of Adelaide, Urrbrae, SA 5064, Australia; 3 School of Chemical Engineering, University of Adelaide, Adelaide, South Australia 5005, Australia; 4 XMPro, North Sydney, NSW 2060, Australia; 5 International Flavour Research Centre, Division of Food, Nutrition and Dietetics, University of Nottingham, Sutton Bonington Campus, Loughborough LE12 5RD, United Kingdom; 6 International Flavour Research Centre (Adelaide), School of Agriculture, Food and Wine and Waite Research Institute, The University of Adelaide, PMB 1, Glen Osmond, South Australia 5064, Australia; 7 School of Engineering, University of Warwick, Coventry CV4 7AL, United Kingdom

**Keywords:** Digital Twins, Internet of Things, precise agriculture, agriculture 4.0, Industry 4.0

## Abstract

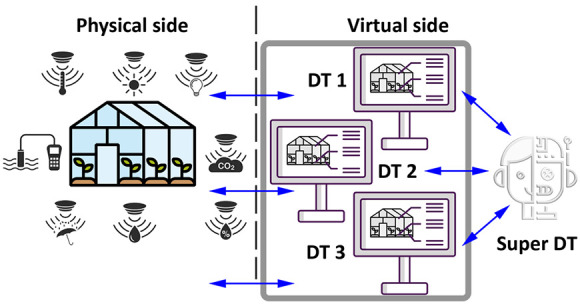

Digital Twins have emerged as an outstanding opportunity for precision farming, digitally replicating in real-time the functionalities of objects and plants. A virtual replica of the crop, including key agronomic development aspects such as irrigation, optimal fertilization strategies, and pest management, can support decision-making and a step change in farm management, increasing overall sustainability and direct water, fertilizer, and pesticide savings. In this review, Digital Twin technology is critically reviewed and framed in the context of recent advances in precision agriculture and Agriculture 4.0. The review is organized for each step of agricultural lifecycle, edaphic, phytotechnologic, postharvest, and farm infrastructure, with supporting case studies demonstrating direct benefits for agriculture production and supply chain considering both benefits and limitations of such an approach. Challenges and limitations are disclosed regarding the complexity of managing such an amount of data and a multitude of (often) simultaneous operations and supports.

## 1 Introduction

The rise of the world population has increased global demands on food production, not only for subsistence but also to support well-being and the shift to a more sustainable lifestyle. This is further compounded by the need to reduce consumption of natural resources and increase agricultural productivity.^1^ Precision agriculture (PA) has emerged subsequently as a more productive alternative, employing advanced agricultural solutions to provide, e.g., exactly the right amount of nutrients at the right moment for the plants. PA has to leverage the collection of a large volume of location-based agricultural data via sensors, enabled by autonomous, disruptive, and data-intensive technologies using Internet of Things (IoT) architecture. This combined approach aims to optimize agronomic inputs such as water, fertilizers, agrochemicals, or soil tillage. Cutting-edge technologies are the Metaverse concept, using virtual-reality space to enable human users’ interaction with a computer-generated environment, and Digital Twins (DTs), aiming at virtual representations of reality.^2^ The latter is reviewed herein.

DTs are defined as “a virtual representation of real-world entities and processes, synchronized at a specified frequency and fidelity, representing the past, present and simulate predicted futures guided by domain knowledge, and implemented in information and operative technology (IT/OT) systems”.^3^ Elemental DT processes include simulation, integration, testing, monitoring, and maintenance, and essential DT units encompass processes, real-world objects, or biological systems created using computational models.^4^ The understanding and manipulation of the virtual replica serves for real-world optimization, resulting in improved efficiencies surpassing limitations, reduced costs, and enhanced decision-making. The physical (real-world) space contains physical assets, sensors, or actuators, while the virtual space includes multiphysics, multiscale, or probabilistic simulation models. Using real-life data collected from past studies, machine learning (ML) models and simulations support data analysis.

**Figure 1:**
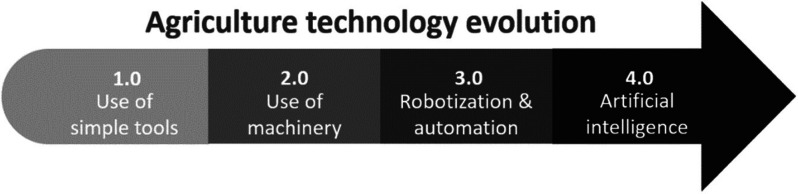
Technological evolution of agriculture along ages.

The interaction and integration of intelligent digital technologies into production, including industrial IoT networks, Artificial Intelligence (AI), Big Data, robotics, and automation, has developed the concept of the fourth industrial revolution, so-called Agriculture 4.0,^5^Figure 1. The benefits and applications of DTs are continuously expanding along different production sectors as industries are willing to discover and explore new production pathways along their capabilities. For example, DTs can be applied to simulate weather patterns, test treatments, or predict productivities oriented toward resource optimization. Not surprisingly, market estimates about IoT predict an increase from USD 7.6 billion to 24.1 billion by 2030, with benefits over USD 1.5 trillion.^6^

**Figure 2:**
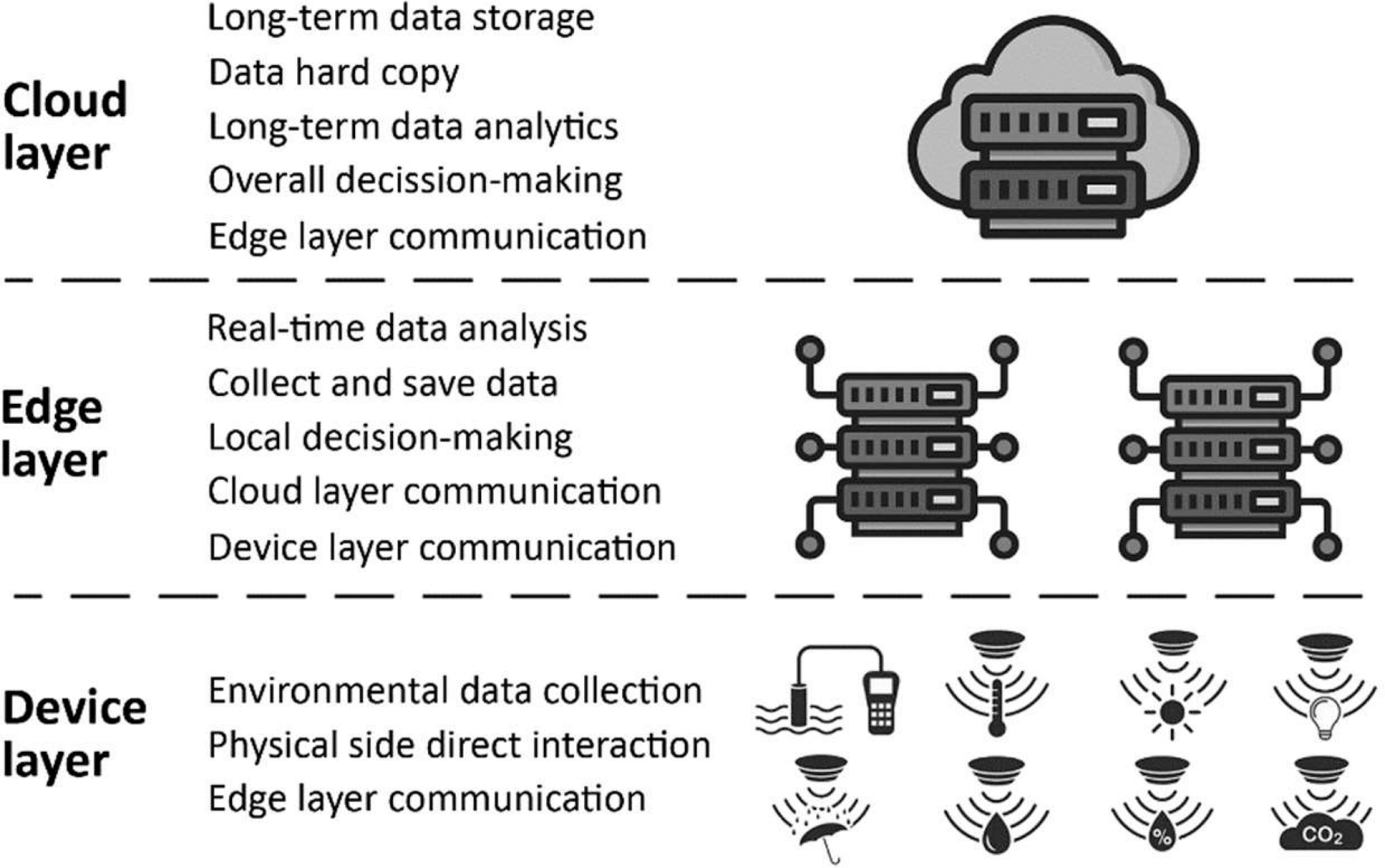
General structure of the IoT platform.

The relationship between an IoT platform and DTs is integral, as they work in tandem to create a comprehensive and interconnected system that enhances monitoring, control, and optimization of physical assets and processes (physical world). A common IoT platform includes three main parts, including the cloud server (backend), connections router, and devices (front end), Figure 2. Each part is equipped with a local decision-making center to minimize bandwidth use, improving reliability, security, and privacy.^7^ The need for real-time interaction makes time the real challenge. Ferrari et al. evaluated the communication delay (round trip time, RTT) due to source–cloud data transfer, setting it to 300 ± 70 ms with a peak below 1 s, concluding that this RTT was still too long for real-time interactions.^8^ This delay time is caused by the interaction of local centers with their own environment using actuators or sensors in a machine-to-machine fashion (M2M), receiving real-time data and preprocessing to ease the cloud through an edge router for a global decision.^5^ M2M connections can be performed directly or indirectly depending on the location of the sensor in or out of the wireless coverage area, respectively, using as many routers as needed to deliver the message.

Local IoT devices are allowed to take local decisions, so they are equipped with a simple deep learning algorithm to compile and process all information provided by sensors or neighboring device interactions, enabling intermediate decision nodes via AI. This architecture mimics the human nervous system,^9^ where with a consumption of 20 W, the brain is composed of 86 billion of neurons whereby information is transferred through synapses using electrical pulses of about 100 mV during 1–2 ms.^10^

The review is divided in three parts, the first being a general description of what DTs are, then an overview of how DTs are orchestrated for agricultural processing, and finally an extended overview of applications of DTs in agriculture with their outcomes and benefits.

## 2 Digital Twins: Architecture and Components

Depending on the complexity and scope, the architecture of a DT can be very different. In a general view, creating and maintaining a DT involves a combination of various machine-to-machine (M2M) technologies that collaborate to replicate, monitor, and analyze physical objects or systems in a digital environment. The emergence of DTs stems from recent development of key computing, communication, and sensing technologies.^11^ Many efforts have been undertaken to provide technical and architectural frameworks for the development and implementation of DTs, i.e., The Digital Twin Consortium’s (DTC) Digital Twin Capabilities Periodic Table^12^ and Reference Architecture.^13^

Architectures may vary according to the following goals: Schleich et al. (2017) introduced a comprehensive architecture to connect physical and virtual twins, focusing on scalability, interoperability, expansibility, and fidelity,^14^ while Alam and El Saddik (2017) proposed a DT for cloud-based cyber-physical systems.^15^

Various DT architectures can be categorized according to the specific purposes or monitoring scopes:^15^ (i) a single DT represents an individual physical object or system, typically used for simple or standalone entities, such as a single machine, equipment, or product, providing a detailed virtual representation of that specific object, enabling monitoring, analysis, and simulation; (ii) a system-level DT represents an entire system or process, encompassing multiple interconnected components and subsystems, offering a comprehensive view while providing insights into the interactions and dependencies throughout the lifecycle, optimizing product performance, monitoring usage, and enhancing maintenance processes; or (iii) a biomass DT represents individuals or groups of living beings in a virtual environment, to be used for production, education, or healthcare. In this review, we focus on agriculture production, whose scope sits between the biomass DT (iii) and a system level production process (ii).

### 2.1 Digital Twin Architecture: Structure of the Data Flow

**Figure 3:**
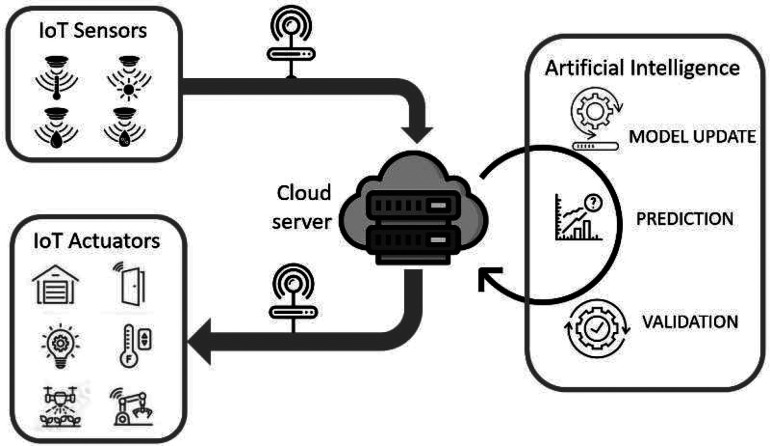
Data flow: data collection, preprocessing, modeling, simulation, analytics, decision support, visualization, and control, ultimately aiding in optimizing farm operations and improving decision-making processes.

In a common DT architecture, sensor data are collected and preprocessed to construct a DT model in the cloud, Figure 3. This data can be both M2M-analyzed to take immediate action and simultaneously used to simulate and construct the optimization model. Through this data flow, the model is continuously updated with new data, so predictions can be validated in real time in response to real events. This iterative process of “continuous monitoring–model update–prediction–validation” allows deep learning, as predictions become more and more accurate. Pedersen et al. highlighted the relevance of interconnections among these steps for the proper functionality of the DT.^16^

The simulation of a DT requires the use of mathematical models to depict and characterize the dynamics of the physical phenomena under assessment, which are fed with data provided by the proper real-time actuators. These models analyze the behavior and relationships of the observed physical phenomena and are validated by real experimental results either in the field or in the laboratory, which inform the DT about the confidence of the previous prediction generating a powerful source of new insight.^17^ The combination of these algorithms in neural networks constitutes an essential tool for AI, to prevent events based on previous data processing, facilitating decision-making, and anticipating actions. DTs are consequently a collection of models built on real-data-bases to assess and predict the behavior of physical processes, designed in a format compatible with automated systems to carry out the corresponding actions.^18^ As technology continues to advance, the integration of DTs with other emerging technologies such as blockchain, AI, and advanced analytics will likely further enhance their capabilities in transforming agriculture.

In the next section, the general components of the DT system architectures are disclosed.

### 2.2 Digital Twin Components

#### 2.2.1 Data Processing and Analysis (I): Artificial Intelligence (AI)

AI enables the comprehension of large volumes of data, which typically could exceed human processing capacities, leading to fast conclusions and decisions that can be executed and carried out by the physical counterpart, in addition to its learning capacities.^19^ Yet with the additional prediction capacity enabled by deep learning, as a result of the storage of previous decisions and derived consequences of actions, DTs enable an intelligent and fast real-life counterpart action against potentially dangerous situations regarding crop health or productivity, irrigation efficiency optimization, and failures of safety mechanisms at different levels (production, postharvest, storage), making immediate M2M corrections to preserve biomass and machinery integrity.^20^

#### 2.2.2 Data Processing and Analysis (II): Internet of Things (IoT)

IoT devices, such as sensors, actuators, and data loggers, are crucial for gathering real-time data from physical objects or systems. In agriculture, IoT devices can measure and capture parameters such as soil moisture, temperature, or images (e.g., using a hyperspectral camera that gives insight into surface chemistry). 5G technology (ultrahigh reliability and ultralow latency) allows DTs for constant monitoring and analysis, taking and controlling precise actions to adapt the physical counterpart to any change in conditions. The contribution of the DT relies on the feedback to the environment, predicting possible situations derived from previous experiences.^21^

#### 2.2.3 Data Collection: Sensors and Actuators

These devices are responsible for capturing and transmitting data from the physical world to the DT. Examples of physical sensors include those that measure critical parameters related to temperature, humidity, pressure, velocity, and material composition; noncontact sensors might capture visible light (camera) or insights into changes in surface chemistry (hyperspectral cameras). Actuators, meanwhile, can enable the DT to influence the physical system as a replication of sensory capabilities. The use of sensors allows the DT to optimize production processes by leveraging previously stored comparative information. Integrating sensors for collecting real-time monitoring information and actuators for taking actions or process corrections enables data-driven analytics to simulate scenarios for optimization purposes, achieving resource optimization and cost savings in various fields.

#### 2.2.4 Communication Technologies

Wireless communication protocols such as message queuing telemetry transport (MQTT) and the constrained application protocol (CoAP) facilitate the seamless M2M transfer of data between IoT devices and the DT. This ensures that the digital representation is continuously updated with real-world data. Effective communication requires a millisecond time frame, currently demanding 5G technology standards. Communication types are sorted by surface communication coverage: (1) RFID (radio frequency identification), a technology that allows multiple objects to be individually identified using radio waves, suitable to be used in, e.g., storage and supply chain monitoring.^22^ (2) Wireless networks from WPAN (short-range connectivity such as Bluetooth) and WLAN (medium-range connectivity such as domestic WiFi) to WWAN (long-range connectivity such as Internet mobile phones). Yet, Low Power Wide Area Networks (LPWANs) have been described as optimal for IoT devices to optimize energy efficiency with long battery life at low cost.^23^

#### 2.2.5 Cloud Computing

Cloud platforms provide the computational power and storage needed to handle the vast amounts of data generated by DTs. Cloud services also support scalability, allowing the DT to grow and adapt when the physical system evolves anytime and anywhere, managing larger data sets without local hardware limitations. Through this approach, the real side of the twin can access and interact with the virtual side from any location and at any time, adapting the situations to environmental changes, such as climatic factors.

Edge computing (deployment of computing and storage resources at the location where data is produced) involves processing data closer to the source (near the IoT devices) rather than relying solely on centralized cloud servers.^17^ This approach can reduce latency and enhance real-time capabilities, which are crucial for applications where quick decision-making is essential.

#### 2.2.6 Virtual Representation: Augmented (AR), Virtual (VR), and Mixed Reality (MR) Technologies

AR, VR, and MR technologies enable users to interact with and visualize DTs in immersive environments. This capability proves particularly useful for training, maintenance, and troubleshooting purposes, from which DTs are derived. For example, the DT enables virtual nature simulations and botanically correct plant models as holograms, similar to Microsoft HoloLens.^24^ Here, the hologram is generated based on real-time data from sensors properly installed in remote key locations, allowing individuals to interact with media enabling physical actions as if interacting with their real-life counterparts. Because of this interaction, tasks can be performed remotely using local robotic systems that mimic human movements, boosting the performance of the DT toward immersive realistic interactions.

#### 2.2.7 3D Modeling, Simulation, Data Analytics, and Machine Learning

**Figure 4:**
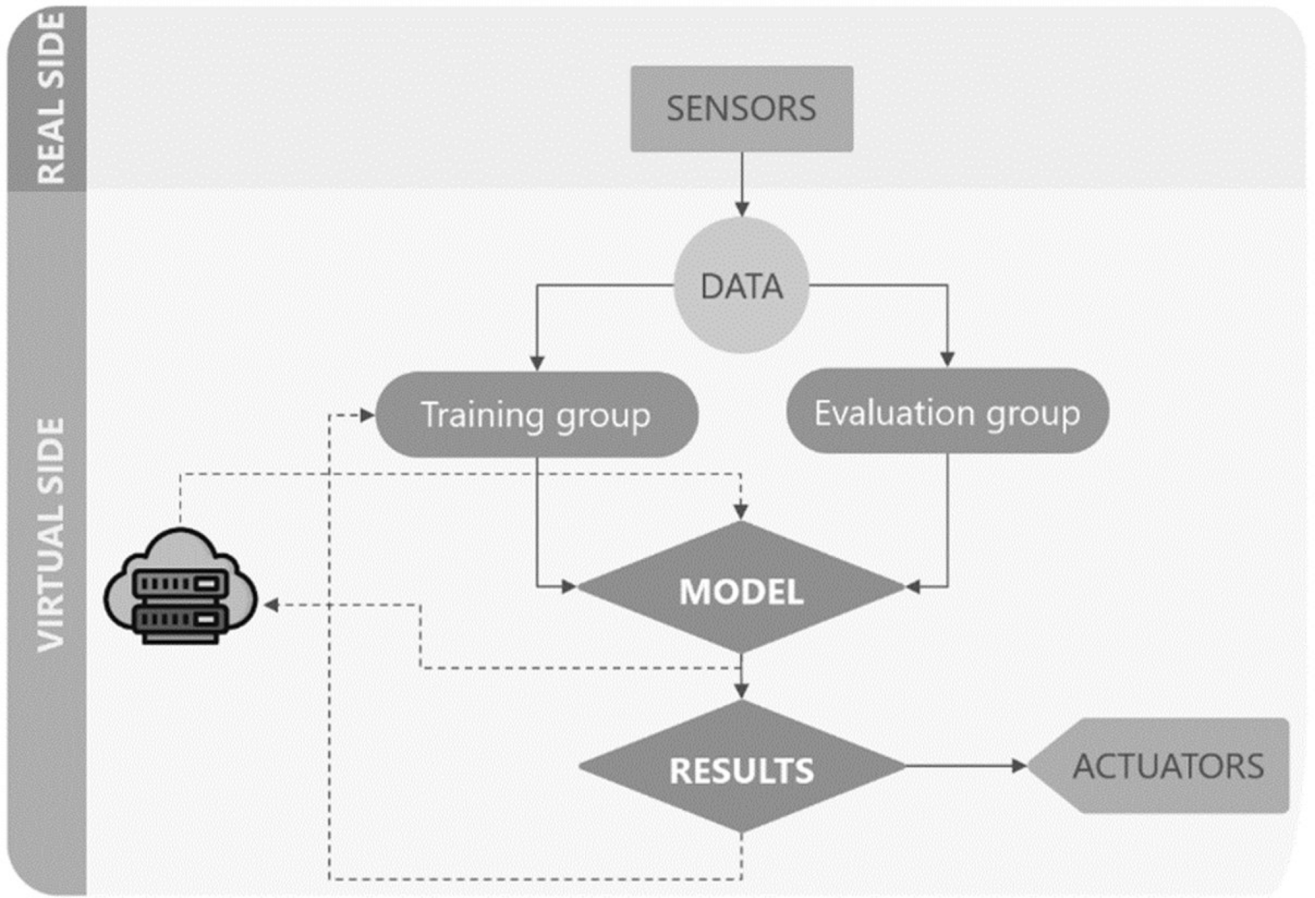
Common machine learning data flow in digital twins.

Advanced analytics, including ML algorithms, play an important role in analyzing the data collected by DTs. These technologies can identify patterns, make predictions, and provide insights that aid in decision-making and optimization. Creating an accurate digital representation often involves 3D modeling and simulation tools.^25^ These tools help in visually replicating physical objects or systems and can be used for testing various scenarios. ML and deep learning (DL) are simulation models, which involve a combination of (i) actual data, (ii) a prediction model, and (iii) a method for modifying and refining the model, Figure 4. The actual data are inserted in the prediction model, which generates a response, conclusion, and/or action This new experience is then considered by the refining model to adjust the model based on real experiences. In an agricultural context, once the model is trained, validated, and verified on the digital side, conclusions about soil and plant health, leaf area, and height can be obtained regarding the physical side. In agriculture, DTs can extend time scales over which the object and its behavior undergo significant variations, such as plant growth.

#### 2.2.8 Safety and Trustworthiness

Besides a strict protection of the DT from cyber threats, robust cybersecurity encryption ensures the integrity and confidentiality of the data associated with DTs. Trustworthiness is taken here globally, meaning the degree of confidence regarding the proper process performance, including safety, security, privacy, reliability, and resilience in the face of environmental disturbances, human errors, system faults, and attacks.^26^ Integrity, reliability, and credibility are also key points when managing key operations to real counterparts, ensuring the authenticity and reliability of data sources. Both virtual and real parts must trust each other to develop a constructive and synergic function, thereby ensuring secure interactions.^27^ This privacy degree is achieved using cryptographic codes and biometric measurements such as ECG (electrocardiogram) and haptic biometrics. Technologies like blockchains facilitate transparency (through agricultural supply chain), security (quality, plant health, and productivity), automation (temperature, irrigation, and plant health), and data storage.^27^

#### 2.2.9 Application Programming Interfaces (APIs)

APIs enable the integration of different software components, allowing DTs to interact with other systems and applications. This interoperability is essential for the seamless flow of data and information. The successful implementation of DTs often involves a combination of these technologies tailored to the specific requirements and characteristics of the physical system or object being replicated. As technology progresses, the capabilities and applications of DTs are likely to expand further.^21^

#### 2.2.10 Physical Entity Model

A physical entity in a DT is not merely a static representation but a dynamic and interconnected digital counterpart that replicates the entity’s geometry, behavior, and characteristics. This digital representation enhances understanding, facilitates optimization, and supports decision-making throughout the entity’s lifecycle.

## 3 Digital Twins in Agricultural Production: Concepts and Benefits

Agriculture is moving toward digitalization through using AI, IoT, and data modeling systems.^28^ A DT carries out the simulation, monitoring, diagnosis, prediction, and control via real-time and accurate digital mapping, e.g., for crops. Agricultural DTs integrate these instruments in cyberspace.

In agriculture, DT technology significantly can increase productivity and efficiency by creating accurate virtual models of crops, livestock, or entire farms.^29^ This technology allows agricultural practitioners to monitor and analyze key factors such as crop growing conditions, soil quality, and climate change in real time to make more precise decisions.

**Figure 5:**
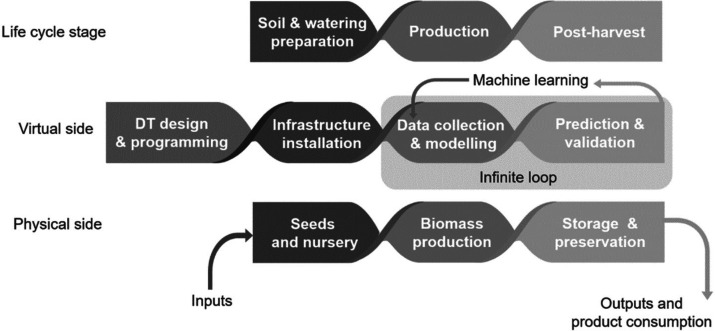
Digital twin interaction along product lifecycle.

This section and the following one are framed by the use of DTs along the lifecycle stages of agricultural production. DTs can be applied from the beginning of a crop production lifecycle up to the final food product, Figure 5. The complexity of agricultural operations asks for a balanced DT design that avoids developing costly dedicated physical prototypes, rather being modular to easily integrate new components for process performance updates.^30^ The DT interacts with the physical twin via monitoring, traceability, compliance, and learning.

### 3.1 Agriculture 4.0: The Smart, Sustainable Way of Digitalization and Automation

Agricultural production in the last three decades has evolved from an artisan business into an automated, highly regulated set of interconnected machines, following trends signaled by the automotive industry in the 1930s and the chemical industry in the 1990s of the past century, respectively. The last step of these so-called agriculture revolutions is Agriculture 4.0 as the fourth agriculture revolution that uses digital technologies toward a smarter and environmentally responsible industrial sector.^31^ This Agriculture 4.0 encompasses digitalization and automation, including Big Data, AI, robots, IoT, and virtual and augmented reality.

### 3.2 Digital Twins along the Agricultural Lifecycle

**Figure 6:**
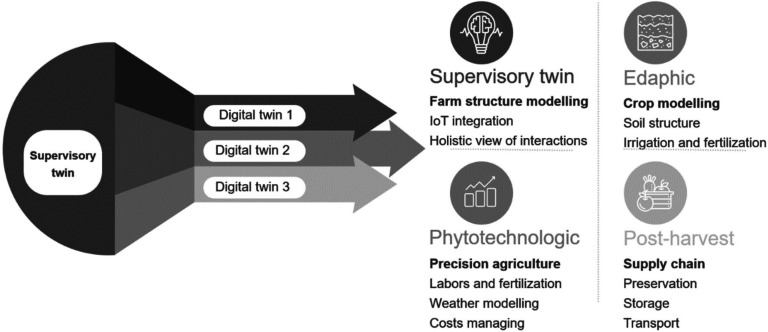
Conceptual structure of multiple DT organizations along lifecycle.

In agriculture and as detailed below, DTs are used to create virtual models of agricultural operations, machinery, and systems. Using sensors and IoT coordinated by cloud computing, DTs simulate and refine farming methods within virtual settings.^32^ As an example, 28 case studies on DTs in agriculture were assessed by Pylianidis et al. highlighting the possibilities of DTs to be applied to revolutionize agriculture.^33^ Yet, agriculture is a complex system with various interaction levels. In an ideal structure, each level would require a specific DT with the corresponding dynamic model to optimize farming operations, thereby reducing environmental impacts while increasing cost competitiveness and productivity, Figure 6. Each DT would be coordinated by a super DT or “Supervisory Twin” suitable to have a holistic view and predict supra-interactions between DTs.^26^

#### 3.2.1 DT Lifecycle Levels

Interaction levels, as depicted in Figure 6, would include the life cycle stages:

(1) An edaphic level, where the soil structure, combined with proper irrigation and fertilization, can be simulated to prevent alterations in agricultural land using IoT real-time data and simulate effects for informed decision-making.^34^
(2) A phytotechnologic level, where labors, fertilizer dosages, and managing costs such as fuel can be improved efficiently and sustainably.
(3) A postharvest level, where the DT can simulate food-related processes such as drying, cooling, transport, and storage degradation.^35^
(4) A farm infrastructure level where levels 1 to 3 are integrated and the DT replicates physical structures on a farm, such as fields, buildings, and equipment, in a virtual environment. This enables farmers to visualize their entire operation digitally.

#### 3.2.2 DT Modeling Functions and Supports

The above-defined levels ask for the following DT modeling functions and supports.

(1) Crop modeling: DTs simulate the growth and development of crops based on various parameters like weather conditions, soil quality, and agricultural practices. This helps in predicting crop yields, identifying potential issues, and optimizing cultivation strategies.
(2) Precision agriculture support: DTs play a crucial role in precision agriculture by providing real-time data on soil conditions, moisture levels, and crop health. This allows farmers or AI to make informed decisions about irrigation, fertilization, and pest control, leading to more efficient resource utilization.
(3) IoT integration: IoT devices, such as sensors and drones, can be integrated with DTs to continuously collect data from the physical environment. This real-time data helps to keep the digital twin updated and facilitates better decision-making.
(4) Climate and weather modeling: DTs can incorporate weather and climate data to simulate how different conditions might affect crops. This helps farmers to plan for potential challenges, such as extreme weather events or changes in temperature and precipitation.
(5) Supply chain optimization: DTs can be extended to model the entire agricultural supply chain, including storage, transportation, and distribution. This allows for better coordination and optimization of the entire food preservation and distribution process.
(6) Decision support systems: By integrating AI and ML algorithms, DTs can provide actionable insights and recommendations; i.e., they can suggest optimal planting times, identify areas requiring additional irrigation, or recommend specific crop varieties based on environmental conditions.
(7) Monitoring and predictive maintenance: DTs can be applied to agricultural machinery and equipment, enabling predictive maintenance, before a breakdown occurs, reducing downtime, and improving overall efficiency.
(8) Data-driven farm management: The data generated and analyzed by DTs contribute to data-driven M2M farm management. Farmers can track historical performance, assess trends, and make strategic decisions to improve productivity and sustainability. In this review, the structure and data flow on different DT integration in agriculture along the lifecycle is overviewed focusing on production.

### 3.3 Sustainability Benefits and Drawbacks of Using Digital Twins in Agriculture

While DTs generally are used in engineering to prevent and respond to critical system failures to maintain product quality, agricultural DTs are more focused on tackling climate change through a proper optimization of scarce natural resources, mitigating the impact of extreme weather, or managing the effects of multiple simultaneous stressors.^36^ DTs propose preventive actions to mitigate or avoid the effects not only in production crops but also in the entire supply chain.

Early detection of potential issues such as disease outbreaks or nutrient deficiencies allows timely interventions through active decision-making before they escalate, thereby maintaining crop health in a sustainable (i.e., lower doses required) and efficient manner in both greenhouses and extended fields.

#### 3.3.1 Sustainability Benefits

**Figure 7:**
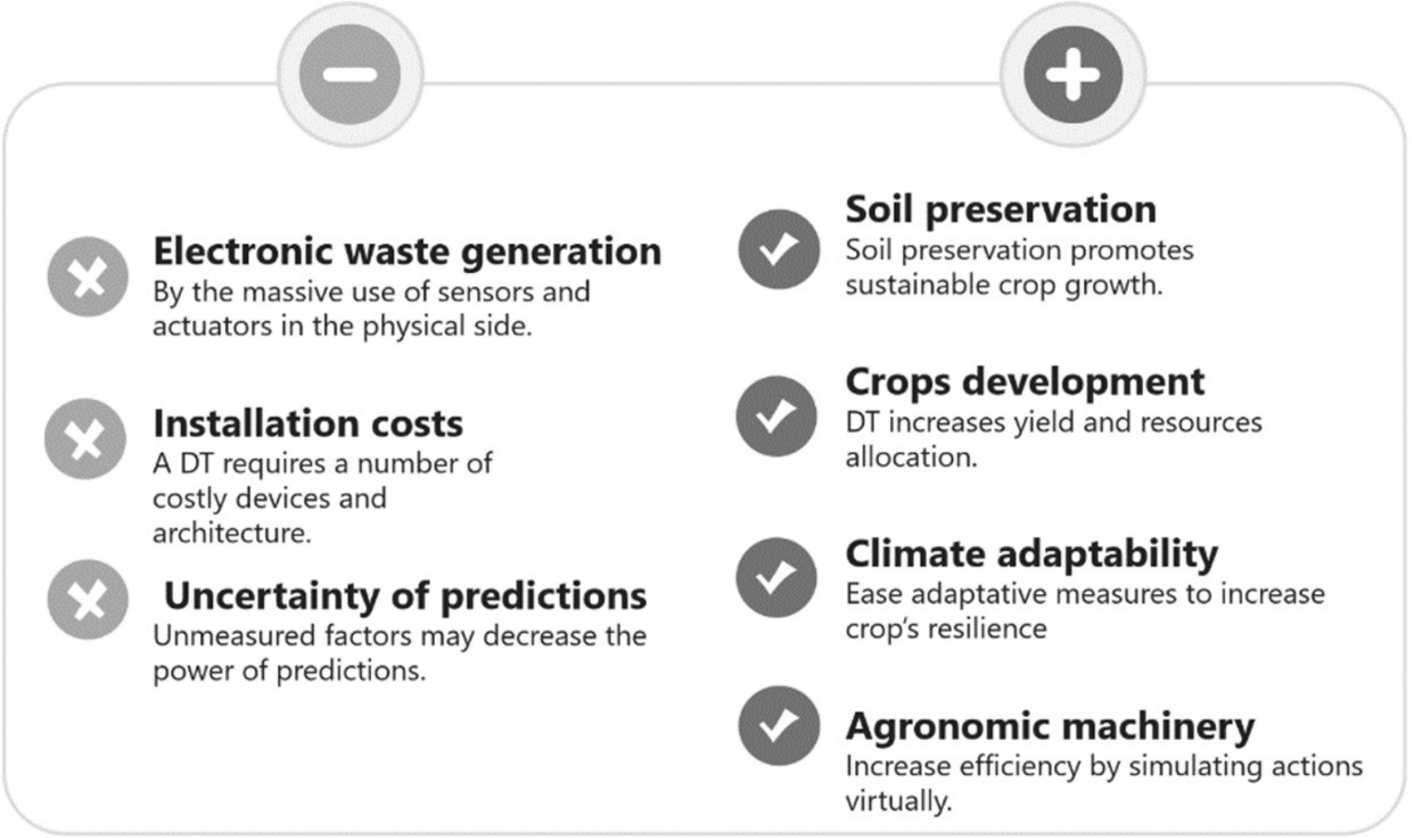
Sustainable benefits and adverse effects of using digital twins in agriculture.

The application of DTs in agriculture can result in business profit and labor savings as well as socioeconomic and environmental benefits. The process intensification derived from the use of DTs includes plant growth intensification, leading to shorter production cycles with optimal use of resources. The application of a DT is initially supposed to result in less resource input and less pollution. A DT equipped with decision-making capabilities has the capacity of taking actions on the environment as the virtual models are transferred to the physical twin. Consequently, this optimization must be evaluated also in terms of the ecological consequences of the selected action,^37^Figure 7.

The aforementioned four-level implementation of DTs in agriculture is expected to deliver sustainability benefits along four key fields within agriculture:

(1) Soil preservation: Soil is the most important resource, as most crops depend on the soil’s health. By assessing soil properties using a DT, nutrients, hydric equilibrium, sustainable fertilization, and irrigation can be planned preventing soil degradation and promoting sustainable crop growth.
(2) Crop development: Virtual simulations of biomass development, mirroring plant growth and behavior, allow decision-making regarding planting, irrigation, and harvesting. This enhances yield and optimizes resource allocation, based on the integration of soil, weather, and nutrient data delivered.
(3) Climate adaptability: Integrating real-time data into a DT allows prediction of crop responses to climate variations. Early modeling enables adaptive measures to be taken to reduce climate-related impacts and improve crop resilience.
(4) Agronomic machinery: DTs allow optimization of machinery use, increasing efficiency and reducing downtime, in addition to the possibility of carrying out virtual tests without compromising real equipment.

#### 3.3.2 Adverse Effects

Adverse effects, however, cannot be excluded and must be considered in a holistic environmental assessment. The use of DTs involves the use of many devices, sensors, and actuators, which might cause electronic waste generation. Implementing better product tracking and return schemes, eased by customers, is crucial for transitioning from the prevailing linear model of “take, make, and throw away” toward Circular Economy.^38^ Innovative business and reverse supply chain models, circular designs, safety for e-waste collectors, and ways to formalize and empower informal e-waste workers are part of the picture.^38^

Additionally, the use of private data, either as individual or as industrial entities, adds a responsible DT layer to a DT system.^39^ Information is continuously transferred including behavior and energy patterns and potential anomalies, which should not be accessible to third parties without the consent of both organizational and user stakeholders. There is a critical need to establish comprehensive privacy policies in DT deployment and design to ensure their responsible and ethical use.

## 4 Digital Twins in Agricultural Production: Application Demonstration along the Lifecycle

Unlike major industry sectors such as chemistry and car manufacturing, the agricultural business is solely conducted through the assembly of industrial machines. Rather, it is at least partly accomplished by using those machines within closed ecological systems (CESs), which create a hierarchy of interlaced systems.

**Figure 8:**
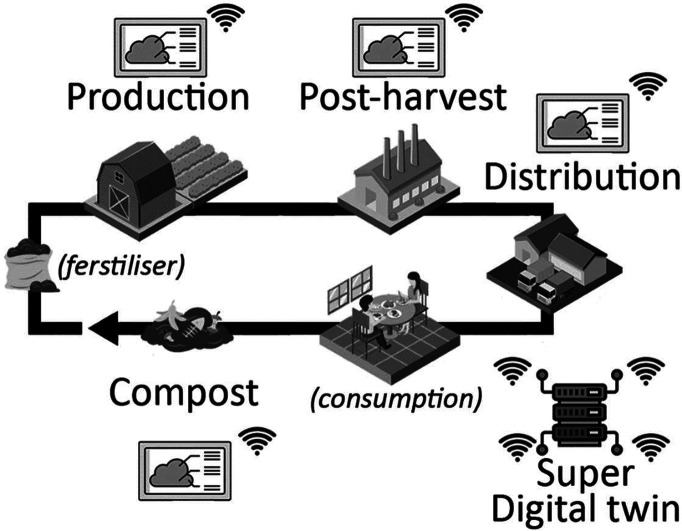
Possible applications of digital twins along the supply chain in agriculture.

The following text is structured in the use of DTs for (i) agricultural machinery, (ii) agricultural resourcing (soil, water), (iii) greenhouses as closed agricultural systems, (iv) hydroponics as closed agricultural systems, and (v) postharvest agriculture, Figure 8.

### 4.1 Digital Twins for the Edaphic Lifecycle

#### 4.1.1 Digital Twins for Soil and Water Management

DTs can simulate different irrigation and fertilization scenarios, enabling farmers to optimize water and fertilizer use, thereby reducing waste while increasing crop yields.^40^ Additionally, DTs can predict the occurrence of diseases and pests, allowing farmers to take more effective local treatment measures, thereby reducing the use of chemical pesticides, protecting the environment, and reducing costs.^41^ Furthermore, DTs can also help simulate the entire agricultural ecosystem. For example, it can be used to assess the impact of different agricultural practices on soil health and biodiversity.^42^ By analyzing soil, climate, and historical data, they can guide farmers to make more effective planting plans, increase yields, and reduce resource waste.

Soil monitoring is central in agriculture and particularly relevant in the early plant growth phase. Insights into the soil can guide the dosage of fertilizers and plant density, with a final impact on the environment, human health, and production costs. A DT was coupled to soil sensors for monitoring moisture (to assess irrigation efficiency), temperature, organic matter, and soil pollutants.^43^ Soil mapping, coupled to AI, provides soil information based on field and laboratory investigations.^44^ Digital technologies used in soil-related DTs include wireless system networks, IoT, edge-computing, local weather-based controllers, and soil sensors. Alves et al. developed a DT for smart water management taking data from temperature and humidity sensors, soil moisture, ambient light, as well as geospatial position sensors, connected to an IoT system, the cloud, and the physical twin.^45,46^ The cloud contains models to simulate the behavior of the soil and crops.

The soil-DT management guides water consumption patterns for reducing water losses^47^ and facilitates maintenance of irrigation management systems.^48^

### 4.2 Digital Twins for the Phytotechnologic Lifecycle

#### 4.2.1 Digital Twins for Weather Modeling

**Figure 9:**
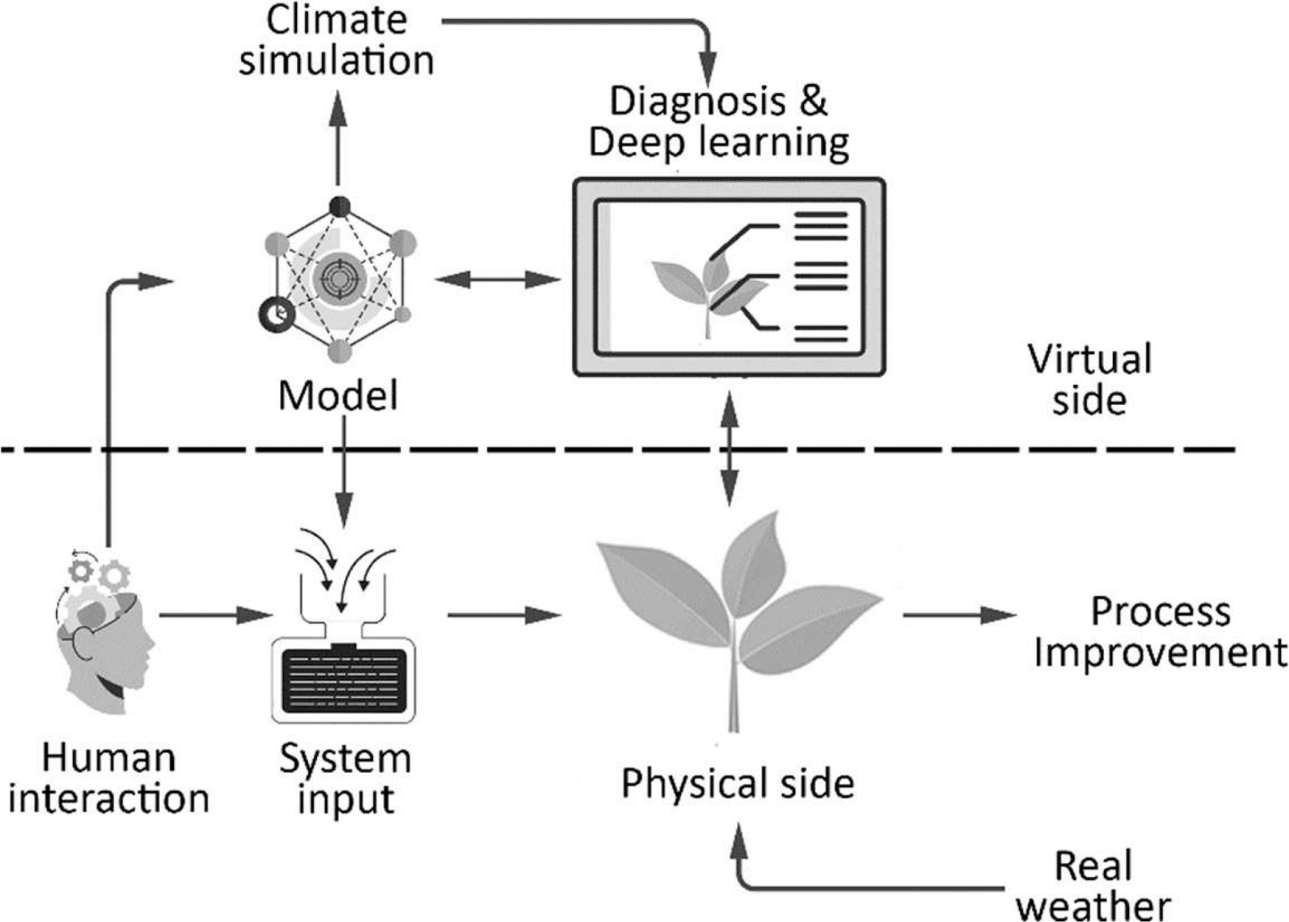
Environment-aware digital twin scheme with weather and climate information.

Numerous reports emphasize the value of a DT for climate adaptation and environmental sciences, marking the next stage of DT use after its application to the manufacturing industry. DTs can serve as interactive models for weather and climate prediction, on a planet scale and for ages.^49,50^ “Environment aware digital twins” (EA-DTs) are weather, climate, and environmental information systems to inform decisions concerning essential industrial and life safety, including cities, ports, flood barriers, energy grids, and transport networks,^49,50^Figure 9. The European Destination Earth (DestinE) initiative developed a DT for climate change adaptation and disaster management on the global scale.^51^ This endeavor allows finally to make better use of renewable resources, water, food, and energy. In Denmark, a national DT has been developed as a hydrological information and prediction (HIP) portal.^52^ Updating of the real-time HIP allows for the integration of submodels. Under the umbrella of the green transition, the European Union is underway to provide funds for DT initiatives. The imperative is to target DT design beyond big data storage, but rather be “fit for purpose” to achieve Earth system simulations and observation capability on a new standard not seen before.^53^ DTs were designed to investigate the impact of weather and climate on urban^54,55^ and natural environments.^56^

Despite the multiple reports on weather and climate DTs and their obvious relation to agriculture, specific agricultural DTs seem to be missing in the literature, as far as we could investigate.

#### 4.2.2 Digital Twins for Cost Management

DTs for lifecycle cost estimation can aid in the early product design stage in manufacturing, yet their application is hindered by the complexity of the processes involved. Uses are reported across diverse industrial sectors, including the optimization and maintenance of railways, impact analysis in the oil and gas industry, and health monitoring.^57,58^ A DT was proposed to overcome this gap via automatic cost modeling using an adaptive data structure and ontologies throughout the product lifecycle.^59^ Another approach to achieve cost-efficiency in DT development has been proposed by modularization via a set of reusable and recomposable DT modules that allow generation of multiple DT variants,^60^ as demonstrated for the space industry. The coapplication of DTs and blockchain technologies helps lifecycle assessment, as practiced in building and construction.^61^

Despite some reports on DT use for cost management, a specific agricultural DT appears to be missing in the literature, as far as we could investigate.

#### 4.2.3 Digital Twins for Agricultural Machinery

**Figure 10:**
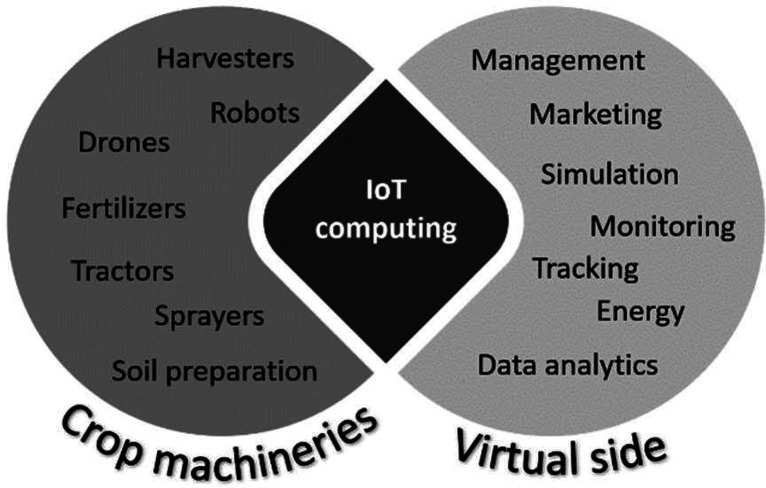
Digital twin architecture for agricultural production.

DTs allow for the automation of agricultural machinery, which serves various purposes, including fertigation, pest treatment, and harvesting. Optimal machinery use can save resources and reduce costs for fuel, fertilizers, and salaries (to substitute manual by robot work), while increasing production volumes and sustainability.^62^ In agriculture, the reliability of machines and predictive maintenance are paramount, particularly during critical harvesting periods. DTs harness the potential of data-driven decision-making to optimize agricultural machinery performance, enabling farmers to prevent breakdowns and minimize maintenance costs. Autonomous machines and robots precisely and reproducibly carry out labors 24/7, ensuring high standards in quality of products,^63^Figure 10. Verdouw et al. developed a DT suitable to track real-time movement of agronomic machines for energy monitoring, economic efficiency, and optimal productivity.^18^ DTs can not only control machines but also allow for M2M programming of the robots to interact without human intervention.^65,66^

### 4.3 Digital Twins for the Postharvest Lifecycle (Food)

The postharvest period encompasses a range of activities such as harvesting, handling, transportation, processing, storage (including drying and/or cooling), and marketing of agricultural products. This phase is crucial for preserving the quality and value of the harvested product as plants cannot withstand any further damage or alterations. Real-time monitoring in an agri-food supply chain DT might interact with these operations, reducing losses and monitoring and optimizing food processing, storage conditions, marketing, and transportation, thereby increasing the robustness and resilience of the chain.^67^ DTs enable the creation of a comprehensive and real-time digital representation of the entire supply chain postharvest. With consumers increasingly concerned about the source, quality, and safety of the food they consume, DTs provide proper tracking and tracign of the purchased products.

#### 4.3.1 Application Demonstration of DT for Postharvest Agriculture (Food)

**Figure 11:**
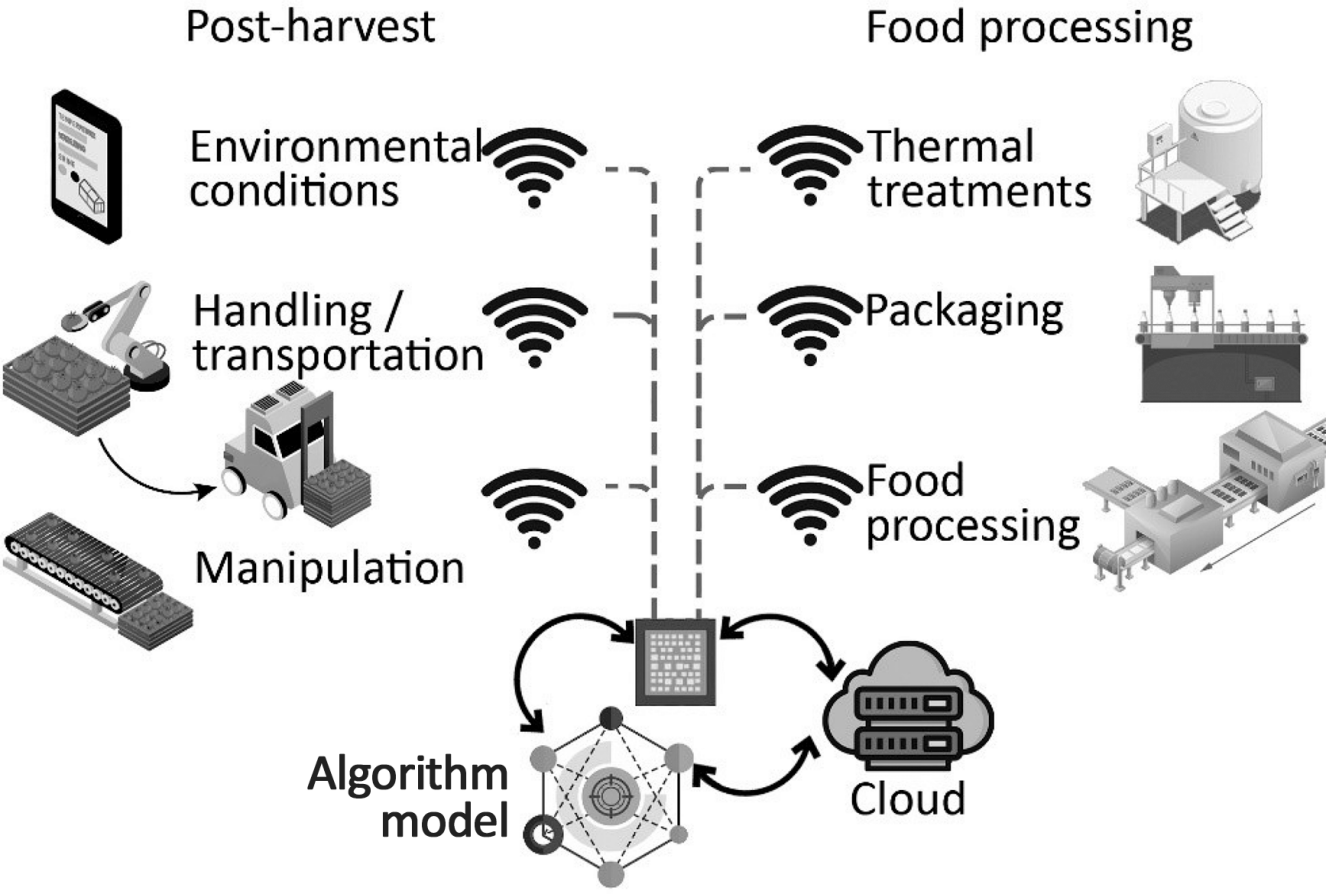
Digital twin architecture in postharvest and food processing optimization.

DTs can track the product along the supply chain, defining traceability parameters and increasing food security. Environmental conditions, handling and transportation, processing, and environmental parameters along postharvest highly influence the quality of vegetables,^68^Figure 11. Verboven et al. reported a DT with the capacity of data collection, IoT with sensor communication, data storage, and big data analytics and a simulation platform with decision supports in the virtual side.^69^ Defraeye et al. applied a DT for assessing the mango postharvest supply chain to simulate biochemical quality changes during this stage.^70^ Factors such as air speed on storage, cold chain length, and delivery air temperature on the fruit quality were measured and assessed in real time by the DT, quantifying fruit quality losses and suggesting proper refrigeration and logistic conditions to reduce food losses. As horticultural products are especially sensitive, the use of a DT is especially advantageous to forecast their shelf life through the cold chain, while customers take greater confidence about product’s quality along the supply chain.^70^ Burgos et al. described a DT based on supply chain analysis, including not only parameters such as production (quality), transportation (i.e., vehicle capacity), warehouses (inventory), sourcing, and shipment (demand) but also insights from customers and suppliers, concluding that DTs can also be used to monitor and correct performance during the food supply chain.^71^ The contribution of a DT in postharvest operations delivers better quality of fresh agricultural products as compared with the current preservation systems, as corrections can be performed before alteration of the product.^72^ Shoji et al. quantified potential quality losses in fruits in the range of 60% on average before being offered in supermarkets, indicating the potential scope of improvement with the application of DTs.^73^ Product quality prediction, shelf life improvement, and cost reduction are the corner stones of DT postharvest contribution.

#### 4.3.2 Industrial Demonstration of DTs for Postharvest Agriculture (Food)

Due to the perishable nature of the final product, the interest in DTs in the food industry has increased in recent years to monitor product deterioration and optimize the shelf life.^74^ Although half of the DT applications in the food industry are related to agriculture, applying methodologies in the way described in previous sections, around a third of the DTs described in food industry refer to industrial food processing.^74^ Key DT-mediated operations include pasteurization,^75^ packaging,^75,76^ entire processing systems,^76−81^ or less extended, optimal product composition and quality.^82^ The remaining 20% is distributed mainly between transportation (8%) and distribution (6%), while the consumption stage remains behind,^74^Figure 11.

Barni et al. suggested good practices for the implementation of DTs in the food industry.^83^ Although the processes are modular and scalable, as they should be, it is recommended to include the entire value chain through dynamic models that allow ML, including variable data from various families to globally channel the prediction. The applications of DTs in the food industry would include all the aspects described in this review regarding production. Yet, most food products have the additional challenge of packaging for protection. The inclusion of intelligent packaging in DTs implies the use of intelligent materials suitable to monitor food conditions and quality state,^84^ achieved by integrating sensors into the packaging^85^ to provide information on variables such as temperature, internal gas composition, pH, moisture, pressure, or vibrations, ensuring traceability of the product along the supply chain.^86^ In this scenario, aiming to avoid over costs, biodegradable biosensors have been developed to detect pathogens or toxins.^87^

### 4.4 Digital Twins for the Farm Infrastructure Lifecycle

#### 4.4.1 DT for Open Agricultural Systems

Laryukhin et al. employed a DT to monitor plant growth and predict outcomes, thus describing the development of a crop.^88^ In addition to the management of critical elements such as land, fertilizer, crop, farmer, etc., the DT was used for economic and resource optimization. In a subsequent step, Skobelev et al. applied this model to wheat production in multiagent modeling to mitigate inaccuracies.^89^ By this approach, the dynamics of the system were described to identify anomalous states and suggest appropriate corrective actions.

Moghadam et al. described a DT for each tree of an orchard using 3D LIDAR and cameras.^90^ In this case, DTs provided real-time condition monitoring and decision support to ease the farmer’s labors. For extensive crops, Machl et al. described a DT referred to a cultivated landscape in order to optimize agricultural transport systems.^91^ The model includes data collection and use in terms of space time, although the time intervals are larger than in other studies.

Angin et al. used a DT for plant monitoring and decision-making using a low-power sensor network and drones, focusing on how new data might generate new insights by employing MobileNet and UNet model algorithms.^92^ Similarly, Jayaraman et al.^93^ and Alves et al.^45^ used DTs for plant monitoring, focusing on irrigation and fertilization in smart farming, including a cloud-based architecture. For the first case, environmental, soil, fertilization, and irrigation data were collected to obtain crop recommendations using the SmartFarmNet framework.

#### 4.4.2 DTs for Greenhouses as Closed Agricultural Systems

Horticulture utilizes indoor production in closed ecological systems (CESs) for reduction of uncertainty caused by weather and soil variability. Greenhouses are a prime technical solution for horticulture as highly controlled, closed environmental systems that can boost plant growth. DTs leverage horticultural parameters through cloud computing, IoT, big data, ML, augmented reality, and robotics. The parameters comprise climate management, irrigation, fertigation, lighting, crop monitoring, disease scouting, harvesting, internal transportation, sorting, and packaging. For example, real-time remote-control eases in-time inspection, while the owner is offshore.

**Figure 12:**
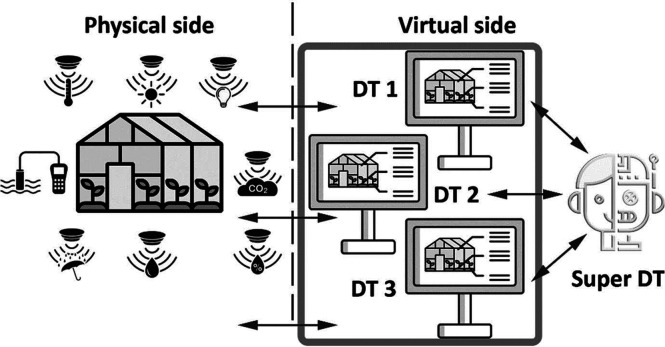
Actors involved in a DT architecture (arrows are data flow).

Greenhouses, as closed environmental systems, ease the integration of Agriculture 4.0, enabling DTs as a fundamental tool to reach productivity optimization. The data flow in a DT between physical and virtual twins is fully bidirectional, synchronizing the digital model with the real-time status of the physical twin. This results in simulations that can be directly implemented into decision-making in crop management and microclimate control for productivity optimization. The basic DT architecture in a greenhouse is outlined in Figure 12, where physical and virtual twins are managed by AI, which hosts the predetermined rules to select for each case the proper alternative. On the virtual side, the DT in greenhouses follows the common architectural compilation of technologies, including IoT; Cloud, Edge and Fog Computing; AI; Robotics; ML; and Big Data Analytics.

##### 4.4.2.1 Building Blocks of Greenhouse DT

Agricultural production DTs embrace data managing and prediction tools and must include additionally (i) architectonic greenhouse facilities; i.e., windows must be suitable to be electronically managed, (ii) sensors and actuators including controllers, i.e., Arduino Uno Microcontroller, and (iii) data storage suitable to be accessed at any time, i.e., using MySQL Database, specifically the phpMyAdmin. Integrating all of these components, the dataflow can be optimized.

**Figure 13:**
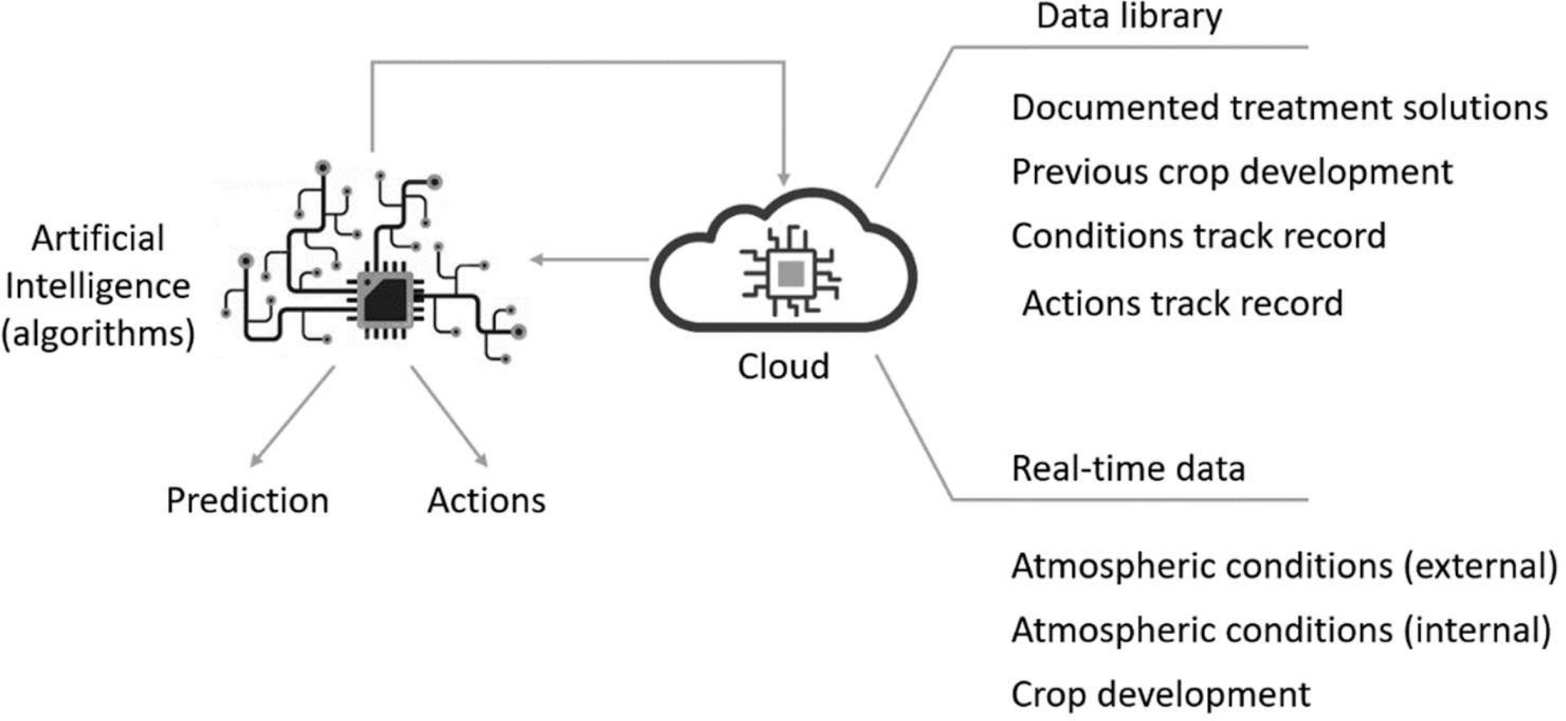
Cloud functions as a data source for AI.

Data can be stored either in the cloud or in a local server, as this is the DT “source of intelligence”, including (i) internal atmospheric conditions as well as external conditions to allow proper balance and interactions, as well as historic previous conditions and treatments (actions taken), (ii) the cloud should be able to receive real-time data constantly regarding (also) crop development, having stored data about previous development, and (iii) a data set of possible treatments and solutions, Figure 13. These data must be available by AI to model, predict, and suggest actions based on the new received data.^94^

##### 4.4.2.2 Digital Displays for Greenhouse DTs

Several system digital display variants are available on the market, which use tailored models for parameter assessment to optimize production decisions.

1. The free-open-source digital display *Energy Plus* can model energy and water.^95^ Heating/cooling, lighting, ventilation, and power receivers are monitored using detailed building physics. *Energy Plus* is compatible with the *OpenStudio* platform,^96^ which expands its capabilities for end users as an interface. The system dynamics can be modeled by defining time steps for thermal interactions between different zones in the greenhouse, allowing manipulation of system dynamics and control strategies with a user-friendly interface.
2. The *Climate Fieldview* digital display uses data-driven decisions to maximize production yields and profits.^97^ The data flow includes collection, storage, and visualization, enabling crop monitoring and measurement of the impact of agronomic decisions on fields.
3. Differently, the *TRNSYS* digital display is a commercial graphical-based application to model dynamic systems such as traffic flow or biological processes besides common evaluation of thermal and electrical systems.^98^ It is divided into two parts; the first reads and processes the input file, solving the system to determine convergence (i.e., using matrix solutions, linear regressions, or interpolations), and plots system variables. The second is a set of around 150 library models of modular components including pumps, turbines, or electrolyzers.
4. The DSSAT digital display monitors and predicts greenhouse productivity via the software *The Rural Technology Transfer Decision Support System*.^99^ This software includes 42 crop simulation models and diverse tools (soil, weather, crop management and experimental data, utilities, and application programs). The models simulate growth, development, and yield as a function of the soil–plant–atmosphere dynamics, requiring daily inputs concerning weather data, soil surface and profile information, and detailed crop management. Simulations include the user option to ask “what if” questions by conducting virtual experiments, including economic risk and environmental assessments for irrigation, fertilizer and nutrient management, climate variability, climate change, soil carbon sequestration, and precision management.
5. The *Agricultural Production Systems sIMmulator* (APSIM) digital display simulates biophysical processes in agricultural systems, focusing on the economic, food security, and ecological outcomes.^100,101^ It incorporates key models required to simulate potential changes in agriculture, with a structure based on plant, soil (i.e., water, N, P, pH), and management modules, which include a range of crops and trees.
6. The *CropX* digital display offers automation and crop management for irrigation and fertilization with accurate forecasts and advanced analysis technologies for agriculture.^102^ It compiles soil, crop, and atmosphere data, suitable to adapt the strategies of optimal cultivation. The system predicts and suggests the proper rate of irrigation, based on soil and weather conditions, according to the crop needs and development stage.

##### 4.4.2.3 Open Digital Displays of Greenhouse DTs

The integration of advanced technologies promoted by Agriculture 4.0 can also be afforded through open platforms, which enable interoperability, collaboration, and innovation across various agricultural processes and stakeholders, yet with limited features as compared with commercial offers. Examples of open data sources are AgriDataSpace (agridataspace-csa.eu) and the Open Ag Data Alliance (openag.io) projects, which provide access to weather information, soil data, crop yields, and market prices. While freely available open-source software is scarce, some examples are FarmOS for farm management (farmos.org) and QGIS for geospatial analysis (qgis.org), which can be integrated using interfaces such as OGC SensorThings (ogc.org) for sensor data exchange and ISO 11783 (ISOBUS) for communication between agricultural equipment. Open designs of open hardware available for modifications and self-customizations can also be found in FarmBot (farm.bot) and Open Source Beehives for beekeeping (fablabbcn.org/projects/osbh-open-source-beehives).

##### 4.4.2.4 Application Demonstration of Greenhouse DTs

DTs together with AI and decision-making programs can optimize growing conditions of a greenhouse, e.g., via strict climate control strategies.^103^ AI and ML models control plant growth based on past experiences, current conditions, and environmental data.^104^ Monteiro et al. proposed a DT for production monitoring in vertical farming, which comprises a model, structure, tasks specifications, assessment of environmental conditions, and decision unit, and outlined its benefits.^105^

Hemming et al. employed DT algorithms to determine climate set points and crop management strategies in six greenhouse compartments during a six month period of cherry tomato cropping with aim to maximizing net profit.^106^ A climate model was combined with a tomato crop model to estimate each compartment’s predicted yield according to growth conditions.

Martin et al. developed a DT to control LED light sources and electronics to create learning models using AI for crops grown in greenhouses, as well as for automotive, streetlighting, and general lighting applications.^107^

The so-called deep learning ResNet^108^ DT was applied to greenhouse tomato crops,^109^ deciphering the interaction between crop quality, environmental factors, and crop management for mango varieties^110^ or potatoes.^111^

Howard et al. proposed a multi-DT based automation of greenhouse production, which allowed garnering of big data through IoT.^112^ Effective communication between the DTs was key with each one in charge of one essential area of the greenhouse. This structure was able to predict future states based on real-time data and databases.

DT simulations prepare for corrective and preventive actions and remote interventions with the corresponding verification and remove constraints concerning place, time, and human observation.^113^ This level of control using the IoT is widely recognized as Agriculture 4.0.

#### 4.4.3 DTs for Hydro- and Aquaponics as Closed Agricultural Systems

**Figure 14:**
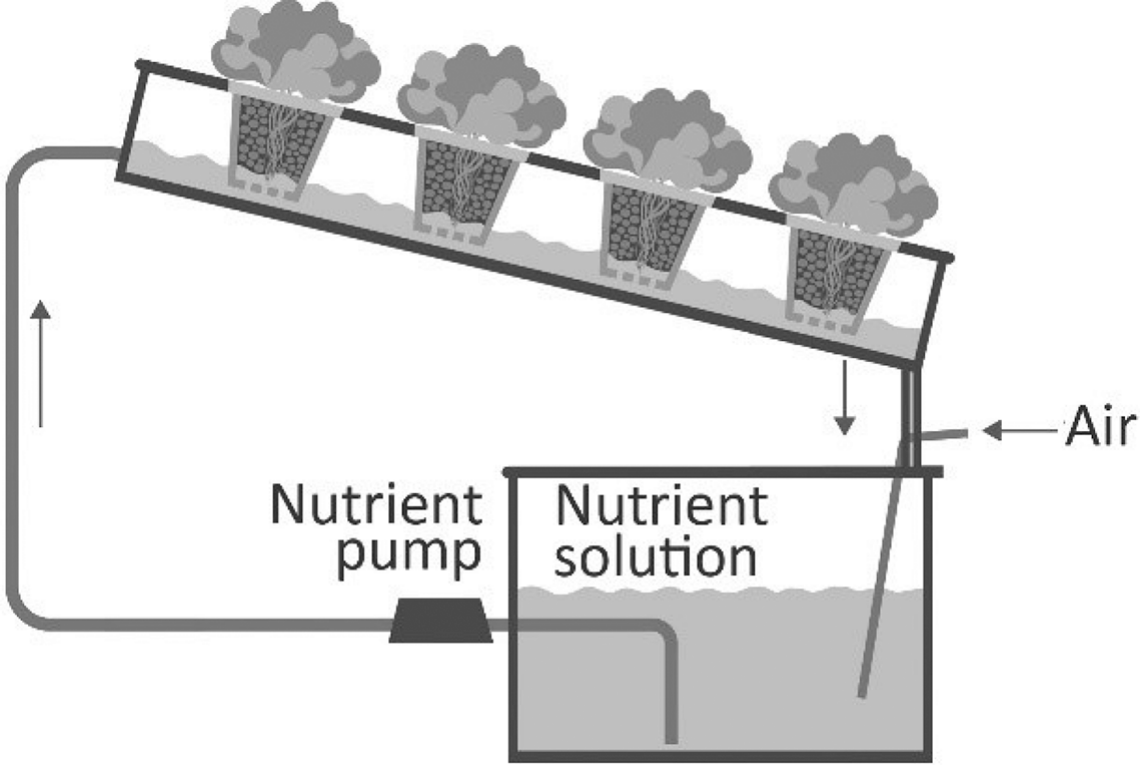
General scheme of hydroponic system parts suitable to be managed by digital twins.

Hydroponics encompasses the methodology of growing plants without soil, where nutrient-rich water is used to deliver the necessary elements directly to the plant roots, while plants are supported by an inert medium like perlite, coconut coir, or rockwool.^114^ Hydroponics adds additional control to crop production in greenhouses to boost plant growth due to direct access to nutrients, Figure 14. Hydroponic systems can be designed for vertical farming and compact spaces. Integrating DTs is a logical addition to this extremely controlled scenario, using the IoT as a building block of smart farming, and using AI algorithms to optimize the productivity according to environmental data, to reduce labor costs and increase profitability. Yet only very few DT studies applied to hydroponics can be found in the literature. A possible cause is that the hydroponics concept itself involves full process control, as technologies such as sensing, remote monitoring, and predictive tools are already part of hydroponics culture.

**Figure 15:**
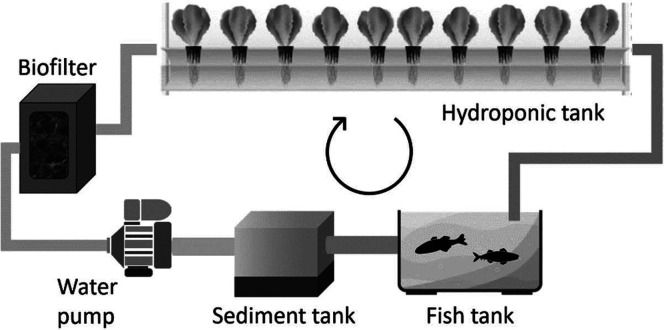
Aquaponic system parts suitable to be managed by digital twins.

Aquaponics combines the use of recirculating aquaculture systems and hydroponics in a circular restorative symbiotic system, Figure 15. Ammonia-rich effluents from aquatic animals are filtered by solid-fixed nitrifying bacteria to convert ammonia into nitrates suitable to be absorbed by root-submerged crops. Reyes-Yanes et al. proposed the use of DTs to acquire data in real time and algorithm-mediated data processing to estimate and optimize the growth rate and fresh crops weight in a restorative aquaponic environment.^115^

##### 4.4.3.1 Application Demonstration of DTs for Hydro- And Aquaponics

Kampker et al. described a morphological framework for a DT to manage a product-service system to support potato harvesting.^111^ To assess the efficiency in customized fields, Tsolakis et al. developed an emulation tool based on the Robot Operating System “AgROS”, suitable to select and import the landscape of a field, adding characteristics of the actual agricultural layout.^65^ A suitable agricultural robot is selected, imported, and tested in a quasi-real-world environment. Alves et al. developed a DT for smart vertical farming by growing a virtual farm to better understand the farm operation and the use of resources and equipment.^45^ Ghandar et al. described a DT and ML to be used for hydroponic systems in urban farming by the implementation of aquaponics (growing plants and fish together in a cyclic nutrient exchange).^116^ The DT modeled the production plan along a 3 month experiment, serving as an approach for an urban farming decision support system.

Jans-Singh presented a DT for an urban-integrated underground hydroponic farm in a World War II air raid shelter.^117^ The originality of the production underground relies on the possibility of physical and virtual side transfer through real-time streams of data, with the possibility to grow plants in adverse conditions. The use of the DT increased productivity per unit area by a factor of 12, while minimizing the energy use, maintaining optimal growing conditions. While the underground conditions provided more stable weather conditions, both the limited ventilation and the light dependence (light emitting diode, LED lights) posed challenges.

## 5 Opportunities, Challenges, and Perspectives

This review presents various digital twin concepts from a general view in agriculture to the specific application of hydroponic systems in greenhouses. Despite being an evolving technology, digital twin technology still faces several technological challenges that need resolution. Nevertheless, it offers numerous scientific and business opportunities across the supply chain. Agriculture 4.0 offers the possibility to monitor and transfer to a digital twin framework many variables such as soil and irrigation, crop, robots and farm machinery, and postharvest food processing.

Digital twins help farmers to increase productivity while efficiently optimizing resources and reducing losses in an Agriculture 4.0 framework, thus reducing economic pressure on the agricultural sector and addressing labor issues. Additionally, digital twins ease research work in exploration of optimal production conditions, by tracking and monitoring crop farm machinery and agricultural and postharvest products or reducing water, chemicals, and energy usage. DTs are expected to become more ubiquitous and accessible, extending even to small- and medium-sized farms.

Different structures and data management sources are commercially available, including technological devices and cloud systems. Digital twins can be integrated throughout the lifecycle, including secondary digital twins managed by a super twin. According to the fixed objectives, different paradigms can be used to construct the next generation of digital twins.

Most of the presented studies are applied to specific needs according to the project, meaning digital twins’ technology is not fully exploited. A comprehensive deployment of state-of-the-art technologies, i.e., AI, advanced statistical and optimization models, big data analytics, and three-dimensional simulations, could lead to a general real-time-based optimization model in agriculture management. Another important trend is the combination of digital twins and IoT technology. By deployment of many sensors and equipment on the farm, a substantial volume of real-time data can be collected, making the DT more accurate and real-time.

Digital twins have the potential to enhance transparency and efficiency in agricultural supply chains by monitoring and simulating the entire process from field to fork. By identifying and addressing bottlenecks and waste, digital twins improve the overall food production and distribution efficiency. The success of digital twin technology also relies on the capacity of establishing circular material flows for fully sustainable production.

On the flip side, one of the main challenges in DTs is their overreliance on automated control. The full control of physical parameters in the virtual counterpart does not exclude potential issues derived from uncontrolled parameters, which in addition can result in irreversible damages.^118^ For some applications, DTs might not be feasible, especially when the physical twin is too complex and requires a great number of resources. DTs face limits when controlling living organisms that are not just a collection of several variables but real complex systems. Besides the obvious knowledge of technology, deep learning, and electronics, the application of DTs in agriculture requires multidisciplinary knowledge of plants growth, diseases, pests, nutrients, etc.

Covering societal demands, not all nations have the capacity to build and use DT-based food-system models due to economic resources and immature infrastructures. The transition to Agriculture 4.0 will require funding to acquire technology for more efficient, sustainable, and secure agricultural practices. DTs need to demonstrate their benefits and a proper return on investment.^119^

The industry uses many sources of simulations to evaluate the performance of a process, with common work packages such as CAD and Aspen offering such capabilities. Singh et al. described several applications of DTs in 13 different industries, including manufacturing, agriculture, education, construction, medicine, and retail.^120^ Yet, the difference when using DTs relies on the constant real-time bidirectional data exchange between the digital and the physical twins, which allows in-time decision-making by taking predictive and/or preventive actions, leading to increased productivity as framed in Industry 4.0.

While in other industries pieces can be monitored given the homogeneity of the production, DT technology in agriculture is still under development given the high variability of each individual crop as a biomass. However, Verdouw and Kruize presented several applications of DTs in different stages of agriculture processing such as crop storage and agriculture machinery.^18^ They focused on livestock monitoring for health detection, identification of pests and diseases in plants, productivity optimization by managing storage availability, and cost-effectiveness evaluation of machinery-mediated treatments. More sophisticated, Monteiro et al. described a DT for vertical farming, collecting data related to temperature, humidity, luminosity, and the relative CO_2_ concentration, properly stored in the cloud and processed using intelligent data analysis resulting in vertical farm optimal production planification.^105^

**Figure 16:**
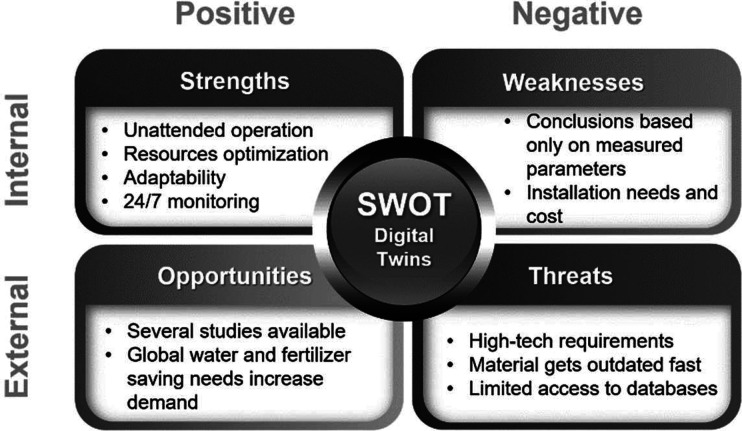
SWOT matrix of digital twin application in agriculture.

Future trends must be based on the strengths and opportunities of DT technology, while future improvements need to counterbalance the weaknesses and threats, as described in Figure 16. Main disadvantages include that the final decisions are based on measured parameters, meaning that other nonmeasured interactions might interfere in the production out of DT control. A complete DT includes installation of several sensors and actuators, which optimally would be wireless for not interfering with agronomic labor, increasing installation costs. The pace of technological evolution is remarkably fast, so devices become outdated in the short term. Additionally, while many applications and databases are currently available as a starting point for a DT, the access to them is mainly limited by the developers due to commercial constraints. Overall, DTs are a key tool for future scientists and engineers to respond to major global challenges (net zero, climate change, water scarcity, etc.), and as future versions of DTs are developed, they will enable scalable system wide agricultural modeling for a diverse range of users in agriculture.

Besides productivity and cost savings, the implementation of DTs in Agriculture 4.0 can improve soil health, namely, its ability to sustain agricultural productivity and protect environmental resources, enabling long-term productivity and avoiding ecosystem degradation. The concept of soil health is extended not only by the physicochemical and biological properties of soil but also by the sensitivity to soil management practices. Through long-term soil and crop monitoring enabled by DTs, agronomic labor such as for localized pesticide application or targeted irrigation is predicted precisely and applied locally when needed, minimizing input usage and maximizing effectiveness avoiding overdosage and soil contamination. DTs help to optimize resource allocation, minimize input wastage, and reduce environmental impacts. Environmental sustainability is achieved by minimizing the use of synthetic (nonrenewable) inputs, reducing soil erosion, and minimizing chemical runoff into waterways, leading to improved soil health, water quality, and biodiversity.
